# DNA Methylation of Alternative Promoters Directs Tissue Specific Expression of Epac2 Isoforms

**DOI:** 10.1371/journal.pone.0067925

**Published:** 2013-07-04

**Authors:** Erling A. Hoivik, Solveig L. Witsoe, Inger R. Bergheim, Yunjian Xu, Ida Jakobsson, Anders Tengholm, Stein Ove Doskeland, Marit Bakke

**Affiliations:** 1 Department of Biomedicine, University of Bergen, Bergen, Norway; 2 Department of Clinical Medicine, Section for Gynecology and Obstetrics, University of Bergen, Bergen, Norway; 3 Department of Medical Cell Biology, Uppsala University, Uppsala, Sweden; Bellvitge Biomedical Research Institute (IDIBELL), Spain

## Abstract

Epac 1 and Epac 2 (Epac1/2; exchange factors directly activated by cAMP) are multidomain proteins that mediate cellular responses upon activation by the signaling molecule cAMP. Epac1 is ubiquitously expressed, whereas Epac2 exhibits a restricted expression pattern. The gene encoding Epac2 gives rise to at least three protein isoforms (Epac2A, Epac2B and Epac2C) that exhibit confined tissue and cell specific expression profiles. Here, we describe alternative promoter usage for the different isoforms of Epac2, and demonstrate that the activity of these promoters depend on the DNA methylation status. Bisulfite sequencing demonstrated that the level of methylation of the promoters in different tissues correlates with Epac2 isoform expression. The presented data indicate that the tissue-specific expression of the Epac2 isoforms is epigenetically regulated, and identify tissue-specific differentially methylated promoter regions within the *Epac2* locus that are essential for its transcriptional control.

## Introduction

Epac 1 and Epac 2 (Epac1/2; exchange factors directly activated by cAMP, also known as cAMP-GEFs) are multidomain proteins that mediate responses via the intracellular signaling molecule cAMP. Binding of cAMP to the cAMP-binding domain activates the Epac proteins, which then act as exchange factors for the small G proteins Rap1 and Rap2 by catalyzing the exchange of bound GDP for GTP (reviewed in [1]). Epac1/2 bind cAMP with about the same affinity as the cAMP dependent kinase (PKA) holoenzyme, suggesting that all cAMP receptors respond to similar physiological concentrations of this messenger molecule [2]. In addition to the cAMP-binding domain, the regulatory domain also contains a DEP module (dishvelled, Egl–10 and pleckstrin homology) that appears to be involved in membrane localization. The catalytic region consists of an exchange activity domain (RasGEF), a Ras-exchange motif (REM) that stabilizes RasGEF domain, and a Ras association domain (RA). Under resting conditions, Epac proteins are inactive due to inhibitory interactions between the regulatory and the catalytic regions. The binding of cAMP relieves the intramolecular inhibition and the catalytic site is exposed to Rap [1].

**Figure 1:**
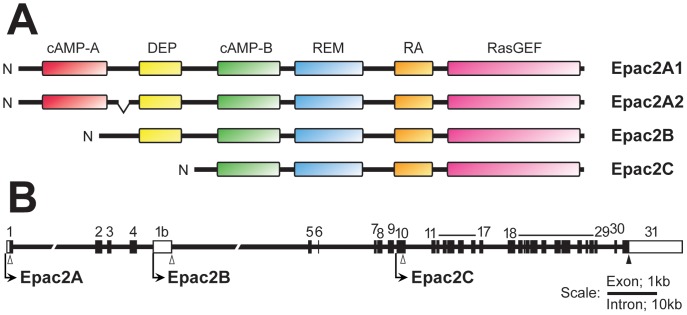
Epac2 protein isoforms and genomic organization of *Epac2*. **A)** **** Schematic illustration of the protein domains of the Epac2 isoforms. The regulatory domain consists of one (Epac2B and Epac2C) or two (Epac2A1/2A2) cAMP binding domains (cAMP-A and cAMP-B) and a dishvelled, Egl–10 and pleckstrin homology domain (DEP; except for Epac2C). The catalytic region consists of an exchange activity domain that catalyses Rap activation (RasGEF), a Ras-exchange motif (REM) and a Ras association (RA) domain. The full-length isoform (Epac2A1) consists of 1011 amino acids (aa), while Epac2A2 is deduced to contain 993 aa. Epac2A2 is identical to Epac2A1, except for an 18 aa deletion corresponding to exon 7. Epac2B consists of 867 aa, and Epac2C of 696 aa. **B)** Genomic organization and alternative promoters at the *Epac2* locus. Coding exons are numbered and illustrated by black boxes. White parts of exons indicate untranslated regions (UTRs). TSSs for the Epac2A, Epac2B and Epac2C isoforms are indicated by bent arrows, while the corresponding translational start sites (ATGs) are indicated by open arrowheads. The filled arrowhead in exon 31 indicates a shared stop codon. The figure is based on the NCBI reference sequence: NC_000068.6, Chr2∶71819344–72094433, and is expanded in 5′-region to include the CpG-island (start; nt 71818997) and in the 3′-region to include the UTR of exon 31 (end; nt 72095526). Note the different scales for exons and introns.

Two separate genes encode Epac1 and Epac2, and alternative promoter usage and differential splicing of *Epac2* give rise to different Epac2 isoforms, three of which have been described in the literature (*i.e.* Epac2A, Epac2B and Epac2C) [3], [4], [5], [6]. Whereas Epac1 is expressed nearly ubiquitously, the expression of Epac2 is restricted, and the different Epac2 isoforms exhibit a confined expression pattern. Epac2B has so far only been detected in the steroidogenic part of the adrenal (i.e. the cortex) [5] and in steroidogenic cell lines derived from the adrenal and testis [7]. Epac2C expression has only been reported in the liver [6]. Epac2A is somewhat more broadly expressed, and this isoform is found in brain, pituitary and pancreatic islets [3], [5], [8], and in pancreatic alpha and beta cells in culture [8], [9]. The differences between the Epac2 isoforms are that Epac2A contains an extra N-terminal cAMP-binding domain (cAMP-A in figure 1A) and that Epac2C lacks the DEP-domain. Epac2B is similar to Epac1 in domain structure (figure 1A). The N-terminal cAMP-binding domain of Epac2A has very low affinity for cAMP and is not involved in cAMP-dependent regulation. Instead this domain has been proposed to direct subcellular localization of Epac2A [5]. In recent years, several physiological roles have been unraveled for Epac2. Epac2A is important for an appropriate pattern of insulin secretion from beta cells [8], [10]. In the brain, Epac2 works together with Epac1 and deletion of both genes, or Epac2 only, leads to impairment in long-term potentiation, spatial learning and social interactions [11], [12]. Moreover, Epac1/2 affects leptin signaling in the hypothalamus [13]. In the liver, Epac2C is involved in bile acid stimulated canalicular formation [14]. At present little is known about the specific roles of Epac2B in the adrenal gland. Although cAMP is important for ACTH-dependent stimulation of steroid hormone biosynthesis, this process is controlled by PKA [7].

We have very limited knowledge about the mechanisms that control the confined expression of the different Epac2 isoforms. Potential promoters for Epac2B and Epac2C have been described in exon 1b [5] and exon 10 [6], but the mechanisms controlling the activity of these promoters are not understood. To gain insights into the mechanisms that regulate the expression of *Epac2* we identified a potential promoter region for Epac2A, and determined that all three promoters exhibited transcriptional activity in a heterologous cell system. Moreover, we established that promoters of *Epac2* are targets for DNA methylation. DNA methylation is an epigenetic mechanism that plays critical roles in differentiation, development and disease. In somatic tissues of mammals, DNA methylation occurs nearly exclusively on cytosines (C) that are followed by guanines (G) (CpG sites). Bisulfite sequencing revealed that CpG sites within the intragenic promoter regions of *Epac2* were subjected to DNA methylation and that the methylation status correlated with promoter activity, such that they were demethylated in the tissue where they are active, but methylated in other tissues.

## Results

### Expression Profiles of Epac2 Isoforms

Three Epac2 protein isoforms are described in the literature [3], [4], [5], [6], and in addition a transcript corresponding to a variant of Epac2A has been submitted to *uniprot.org* (A2ASW3 protein entry number) (figure 1A and figure S1). This latter isoform has to our knowledge not yet been identified at the protein level, but would, in theory, upon translation be identical to Epac2A except that exon 7 is spliced out (figures 1A and S1). In this paper, the isoform that was previously described as Epac2A is referred to as Epac2A1, and the novel Epac2A variant is referred to as Epac2A2. Transcription of Epac2A is initiated from exon 1 [3], and transcription of Epac2B from exon 1B ([5], figure 1B). This isoform thus lacks cAMP-A found in Epac2A. Epac2C is also devoid of the N-terminal cAMP-A as well as the DEP domain (figure 1A, [6]). Transcription start sites (TSSs) for Epac2C have been identified in exon 10 ([6], figure 1B).

**Figure 2:**
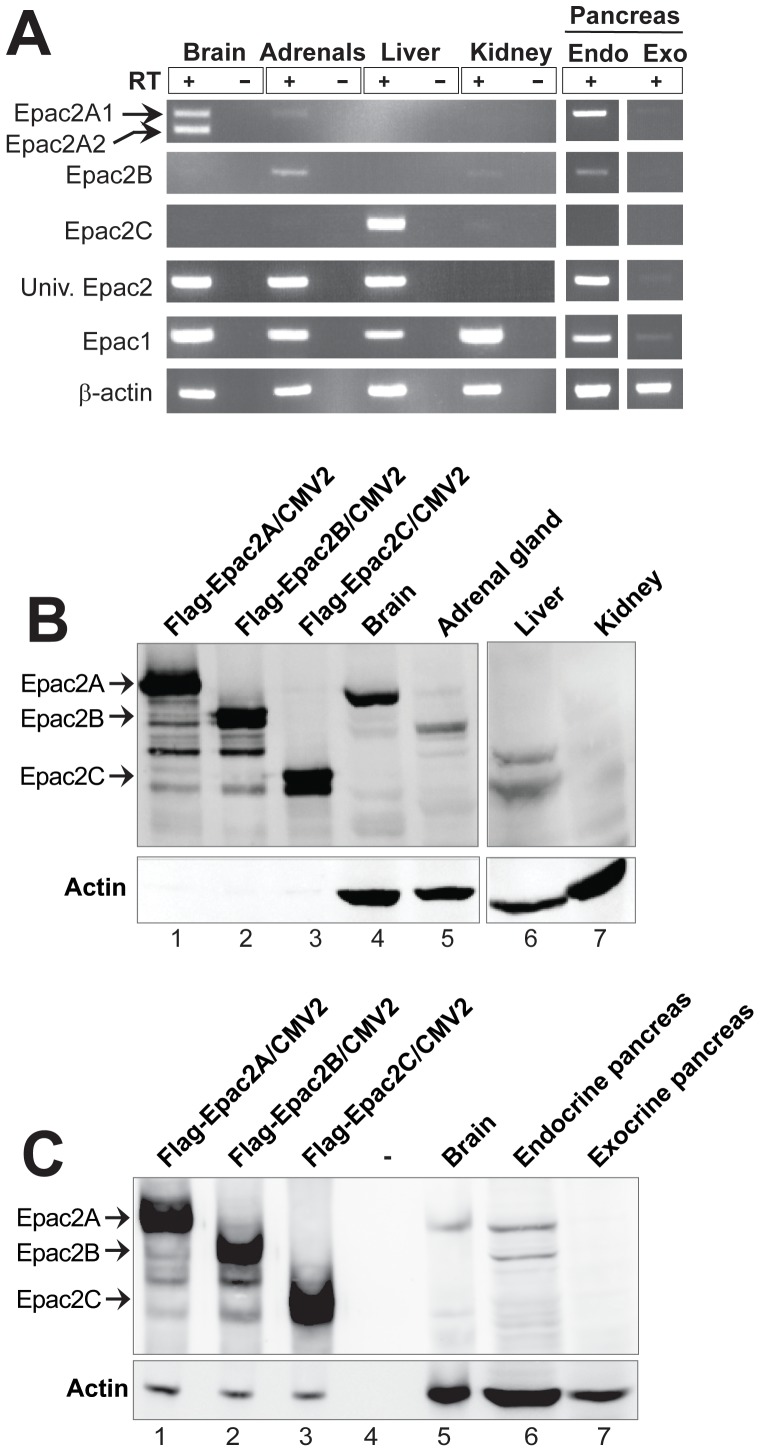
Expression of Epac2 isoforms. **A)** mRNA was prepared from mouse brain, adrenal glands, liver, kidney and endocrine and exocrine pancreas, converted to cDNA, and RT-PCR was performed with primers as specified in table 1. +/−; presence and absence of reverse transcriptase (RT). **B–C)** Immunoblotting with Epac2 antibodies was performed on protein extracts prepared from Cos1-cells overexpressing Flag-tagged Epac2A, Epac2B or Epac2C (20 μg; B, C; Cos1-cells do not express endogenous Epac2 [9]), and extracts prepared from mouse brain, adrenal gland, liver and kidney (250–300 μg; B) as well as from brain and endocrine and exocrine pancreas (250–300 μg; C). The migration of the different isoforms is indicated by arrows. Immunoblotting against actin is shown below each blot. The apparent absence of actin in the Cos-1 cell extracts in B is explained by a very short exposure time of the blot (as only 20 μg of Cos-1 extract was loaded on the gel compared to 200–250 μg of tissue extract).

Due to the low expression levels of Epac2 in most tissues and the lack of well functioning antibodies, the expression profile of Epac2 has mainly been determined by Northern blotting and reverse transcriptase (RT)-PCR. The applied RT-PCR primers have not, however, been designed to distinguish between the different isoforms. To examine the potential presence of more than one Epac2 isoform within the known Epac2 expressing tissues, primers that specifically amplify each isoform were designed (see table 1 for primer sequences). Both Epac2A isoforms (Epac2A1 and Epac2A2) were detected in brain, but neither Epac2B nor Epac2C (figure 2A). Epac2A2 was not expressed in any of the other tissues examined and might be a brain-specific isoform. The adrenal gland was found to express mainly Epac2B, although a faint band appeared with the Epac2A1 specific primers (figure 2A). Only Epac2C was amplified from liver mRNA (figure 2A). Both Epac2A1 and Epac2B were detected in the endocrine pancreas, whereas the exocrine pancreas did not express any of the Epac2 isoforms (figure 2A). As expected, a universal Epac2 primer pair produced products in all Epac2 expressing tissues. RNA from the kidney was used as a negative control as this organ has been reported to expresses only Epac1 [3]. We did however, detect very faint bands for Epac2B and Epac2C in this organ (figure 2A), but this was not confirmed by immunoblotting. Epac1 was detected in all tissues examined (figure 2A), presumably because of the general expression of Epac1 in endothelial cells [15], [16]. Sequencing verified that the PCR-products had the correct identity. Immunoblotting confirmed the RT-PCR results (figure 2B-C), and generally these results are in agreement with published literature on Epac2 expression in mice [5], [6], [8]. Importantly, however, the identification of Epac2B in endocrine pancreas is novel.

**Table 1:** Overview of primers employed in RT-PCR assays.

Target region	Direction	Sequence (5′–3′)	PCR Size (bp)	Target exon/UTR
Epac2A1/2	F	CACGCGGCTGAAAGGAGTTAA	561	Exon 2
	R	ATCATGTGGGGAGCTCGAGAGA		Exon 8
Epac2B	F	TGGCTGCTTACTGGATGTCTGAGAA	274	5′UTR of exon 1b
	R	ATCATGTGGGGAGCTCGAGAGA		Exon 8
Epac2C	F	GCCTCCATGTTTCCCGCAG	228	5′UTR of exon 10
	R	GTCATCCACAGTCCTCTGGCCA		Exon 11
Epac2 Univ.	F	ATCTACGACGAGCTCCTTCATATTAAA	176	Exon 11
	R	GACTACATTCACGGATCCTTTCAGA		Exon 13
Epac1	F	GAAAATGGCTGTGGGAACGTATCT	383	Exon 16
	R	AGCTGCTCAGGGTGTGGGGT		Exon 18
β-actin	F	AGGCCCAGAGCAAGAGAGGTATC	264	Exon 2
	R	AGGCATACAGGGACAGCACAGC		Exon 3

### The Epac2 Gene Contains Alternative Promoters

**Figure 3:**
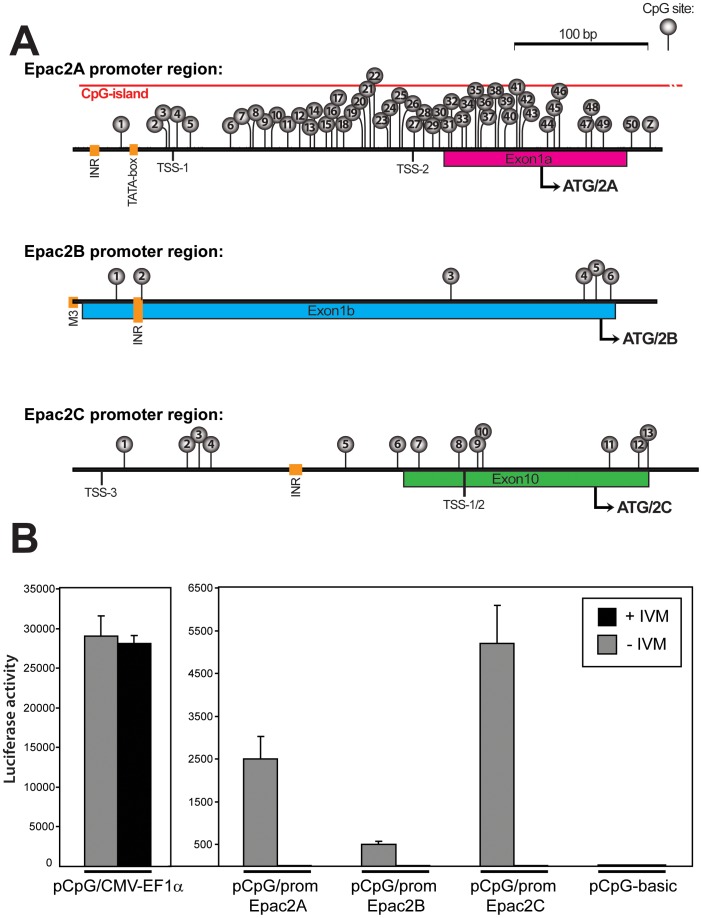
The predicted promoters regions of *Epac2* contains CpG dinucleotides and hold transcriptional activity. **A)** Schematic drawing of the predicted promoter regions of *Epac2*. The promoter region directing expression of Epac2A (chr2 71980880–71981553; upper panel) contains a potential INR- (initiator) like element, a TATA-box and two putative TSSs. A CGI with 51 CpG sites (indicated by grey lollipops) covers the promoter region. The promoter region directing expression of Epac2B (chr2 72054237–72054766; middle panel) contains a putative INR element and a M3 element. This region contains 6 CpG sites as indicated by lollipops. The promoter region directing expression of Epac2C (72179738–72180207; lower panel) contains three alternative TSSs (TSS1–3), as well as an INR element as also described previously [6]. Transcription is initiated from TSS1/2 and not TSS3 [6]. This region contains 13 CpG sites. The translational start sites are indicated by bent arrows. To identify novel potential promoter regions in *Epac2* the sequences corresponding to potential promoter regions were analyzed by the FlyBase eukaryotic promoter prediction computational tool (http://flybase.org) and rVISTA evolutionary conservation analysis [17]. The promoter regions shown correspond to the genomic regions that were analyzed by bisulfite sequencing in figures 4–6. Note that the CpG-site marked with a “Z” (upper panel) is included in primer sequence and therefore not included in the BSP analysis). **B)** The reporter gene plasmids containing the *Epac2* promoter regions (pCpG/prom-Epac2A, pCpG/prom-Epac2B and pCpG/prom-Epac2C; 200 ng), the positive control vector pCpG/CMV-EF1alpha (200 ng) and the pCpG-basic vector (containing no promoter/enhancer; 200 ng), were transfected into Cos-1 cells. Before transfection the plasmids were either *in vitro* methylated by the *Sss*I CpG methylase (black bars) or left untreated (grey bars). IVM; *in vitro* methylation. The luciferase activities are presented as average +/− stdev, n = 9.

To identify novel potential promoter regions in *Epac2,* genomic sequences corresponding to regions around the alternative translational start sites were analyzed by the FlyBase eukaryotic promoter prediction computational tool (http://flybase.org) and the rVISTA evolutionary conservation analysis tool [17] (figure S2). rVISTA analyses identified highly conserved regions in all three putative promoters with a conservation of 76–79% between mouse and human (mm10 base genome was compared to human hg19) (figures S1 and S2). The combined conservation and promoter prediction analysis identified a conserved promoter region for Epac2A1/2 that contains initiator (INR) elements and a classical TATA box, (figure 3A, upper panel). The predicted promoter region of Epac2B contained several INR-elements, and also a M3 element (typically enriched in TATA-less promoters) (figure 3A, middle panel). The Epac2C-promoter was found to contain alternative TSSs (TSS1–3), as well as several INR sites and a TATA box, as also described previously (figure 3A, lower panel [6]). Transcription from this promoter is mainly initiated at TSS1/2 [6].

None of the Epac2 promoters have previously been functionally evaluated. Luciferase assays were therefore performed to experimentally examine the transcriptional potential of these promoters. Fragments containing the promoter regions were inserted into the pCpG-basic vector, and as shown in figure 3B, all three promoters enhanced reporter gene activity when compared to the promoter-less pCpG-basic vector. The Epac2A and Epac2C promoters were considerably more active than the Epac2B promoter (figure 3B). This might be explained by the lack of potential tissue specific factors in this heterologous cell system. To identify potential CpG sites in the *Epac2* promoter regions, the sequence corresponding to the *Epac2* gene region (exon 1–32, including upstream and downstream regions of 3 kb; mouse chr2 71978215–72260469) was analyzed with the *cpgplot* software by employing default CGI prediction settings of; length >200 bp, GC content >50% and observed CpG/expected CpG >0,60 [18]. One CGI of 548 bp was found in *Epac2*. This CGI contains a total of 60 CpG-sites and coincides with the Epac2A-promoter region (figure 3A, upper panel and figure S3). The Epac2B-promoter contains 6 CpG sites across a region of 419 bp (figure 3A, middle panel) and the Epac2C-promoter 13 CpG sites in a region of 446 bp (figure 3A, lower panel). The presence of CpG sites in the *Epac2* promoters raised the possibility that their transcriptional activities might be regulated by DNA methylation. The promoter containing reporter-plasmids were therefore methylated *in vitro* prior to transfection into Cos-1 cells. The pCpG vector, including the CMV promoter/EF1alpha enhancer elements, is devoid of CpG sites, and as expected therefore, luciferase expression from pCpG-CMV was not affected by *in vitro* methylation (figure 3B). In contrast, reporter gene expression driven by the *Epac2* promoter regions was nearly abolished as a consequence of methylation, suggesting that the DNA methylation status is important for their activity (figure 3B).

### The CGI of the Epac2A-promoter is Demethylated in all Tissues Examined

**Figure 4:**
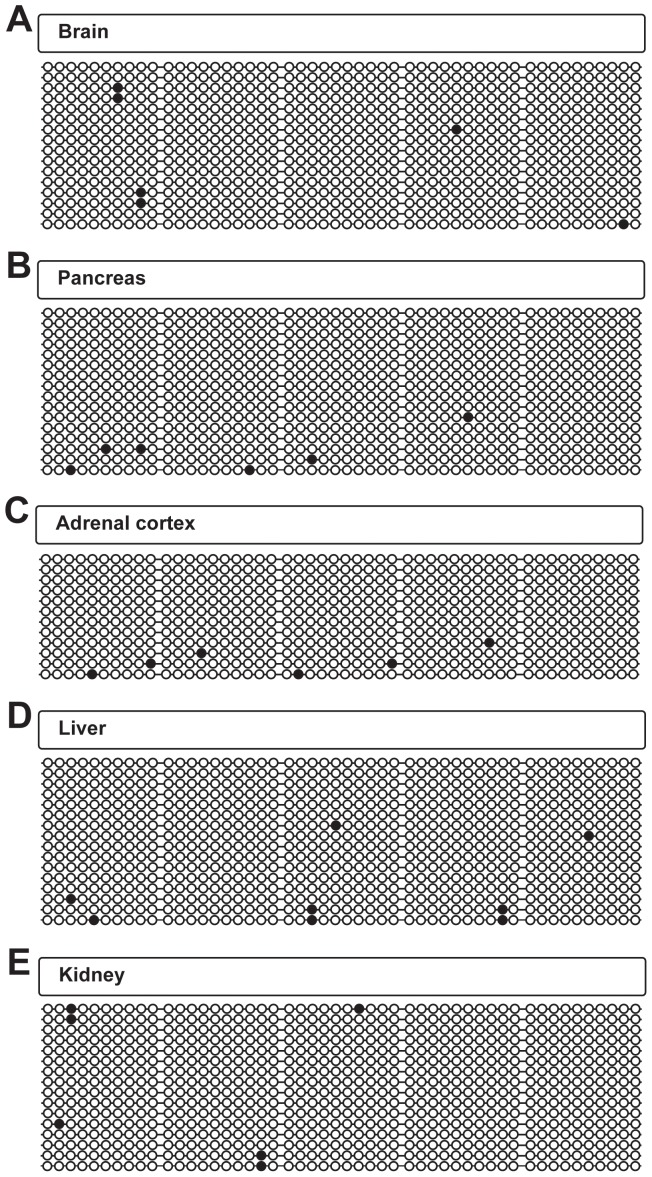
The CGI of the Epac2A-promoter is demethylated in all tissues examined. Bisulfite sequencing analyses of the *Epac2* A-promoter (containing 50 CpG sites across a region of 441 bp, spanning nucleotides 71980937–71981377 of chr2). DNA was prepared from brain (A), pancreas (B), adrenal cortex (C), liver (D) and kidney (E). Bisulfite sequencing was performed on pooled DNA; from 7 mice in A–B and D–E, and from 8 mice in C. For all tissues, the percentage of methylated CpG sites was ≤1%. The total number of analyzed clones were: 32 in (A), 30 in (B), 12 in (C), 35 in (D) and 34 in (E). Each horizontal line represents one analyzed clone. Black circles: methylated CpG sites, open circles; demethylated CpG sites. Representative clones are shown for A–B and D–E.

To determine whether the expression pattern of the different Epac2 isoforms is reflected in the methylation status of the promoters, we determined the level of methylation in selected mouse tissues. The methylation status of the Epac2A-promoter was determined using primers that amplify 50 CpG sites over a region of 441 base pairs (bp), which make up the majority of the CpG-sites in the CGI (figure 3A, upper panel). Bisulfite sequencing revealed that the Epac2A-promoter was virtually completely demethylated in all tissues tested, both in Epac2A expressing tissues (brain and pancreas, figure 4 A-B), in tissues that express other Epac2 isoforms (adrenal cortex, liver and pituitary figure 4 C-D and data not shown) as well as in non-expressing tissues (*e.g.* kidney) (figure 4E and data not shown). This finding is in agreement with whole genome studies that repeatedly have demonstrated that the majority of CGI-containing promoters are demethylated regardless of the expression level of the corresponding gene [19], [20], [21].

### The Epac2B and 2C Promoters are Hypomethylated in Tissues Expressing only Epac2B or Epac2C, Respectively

**Figure 5:**
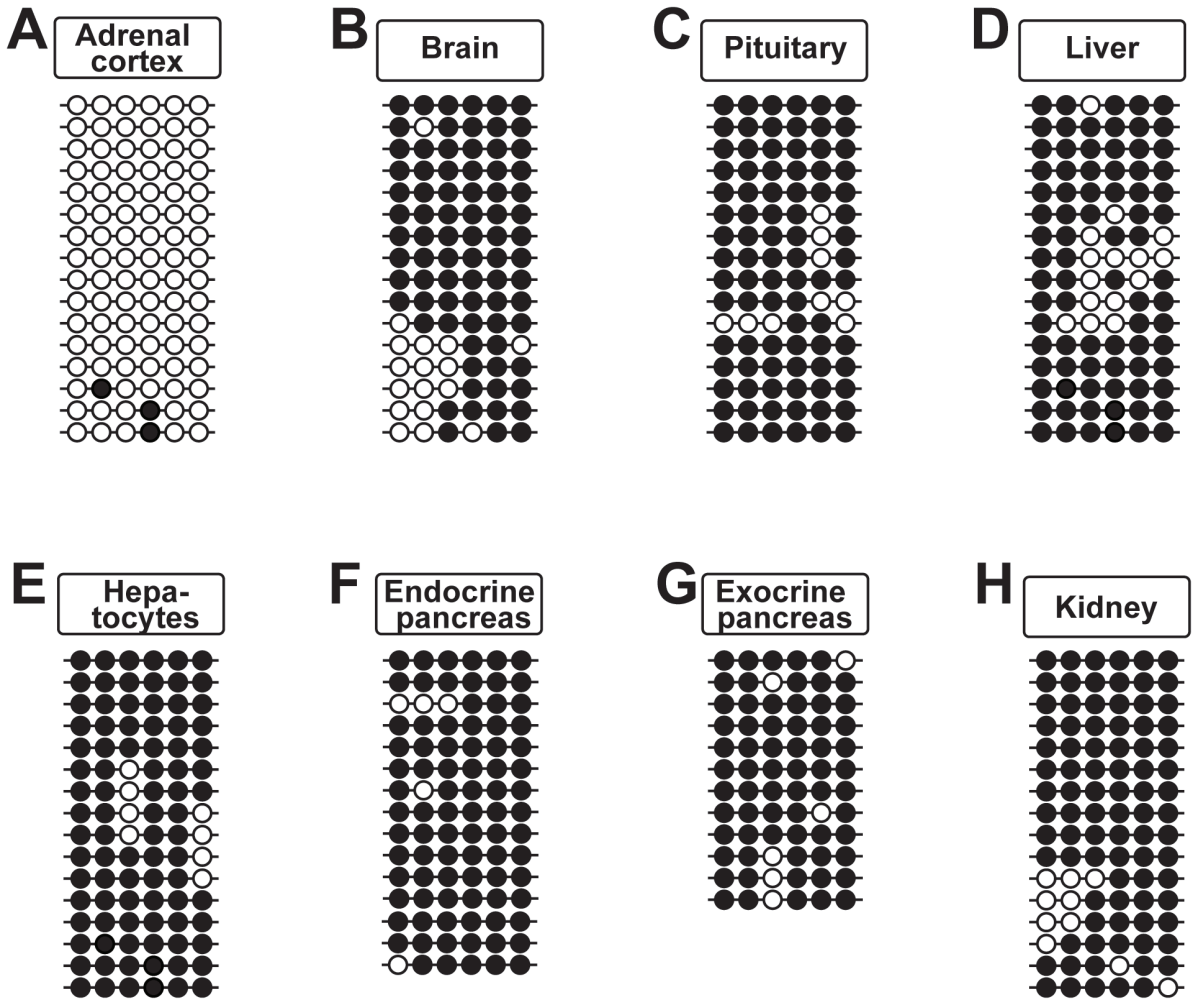
The Epac2B-promoter is hypomethylated in the adrenal gland. Bisulfite sequencing analyses of the *Epac2* B-promoter (containing 6 CpG sites across a region of 438 bp, spanning nucleotides 72054618–72055055 of chr2). Genomic DNA was prepared from adrenal cortex (A; 8 mice), brain (B; 7 mice), pituitary (C; 5 mice), liver (D; 7 mice), freshly isolated hepatocytes (E; 3 mice), endocrine pancreas (F; 5 mice), exocrine pancreas (G; 5 mice) and kidney (H; 7 mice). Bisulfite sequencing was performed on pooled DNA from the number of mice indicated above. The overall percentage of demethylated CpG sites and the number of clones analyzed were (number of clones in parentheses): 94% (39) in (A), 20% (46) in (B), 7% (28) in (C), 19% (46) in (D), 13% (58) in (E), 9% (38) in (F), 8% (12) in (G) and 23% (41) in (H). Each horizontal line represents one analyzed clone. Black circles: methylated CpG sites, open circles; demethylated CpG sites. Representative clones are shown for A-F and H.

To establish the methylation pattern of the Epac2B-promoter, primers were designed to amplify 6 CpG sites across a region of 438 bp (figure 3A, middle panel). In the adrenal, Epac2B is only expressed in the cortical portion [5], and laser capture micro-dissection was used to obtain pure cortical tissue. In agreement with the specific expression of Epac2B, DNA isolated from adrenal cortical cells was hypomethylated across the putative Epac2B promoter (figure 5A) compared to tissues that do not express this isoform. This was true both for tissues that express predominantly Epac2A (brain and pituitary, figure 5 B-C), Epac2C (liver and primary hepatocytes figure 5 D-E) and tissues expressing Epac1 (exocrine pancreas and kidney; figure 5 G-H). As shown in figure 2C, the endocrine pancreas expresses both Epac2A and Epac2B at similar levels, possibly indicating cellular co-expression of both isoforms. Intriguingly, the putative Epac2B promoter was found to be hypermethylated in the endocrine pancreas (figure 5F). Of a total of 38 clones analyzed by bisulfite sequencing, none contained more than 3 demethylated CpG sites (overall only 9% of the CpG sites were demethylated). The intensity of the protein band corresponding to Epac2B (figure 2C) indicate that it is expressed in a substantial number of the islet cells, thus potential hypomethylated clones would most likely have been identified if the Epac2B promoter was hypomethylated in this tissue. These results indicate therefore, that a hypomethylated status is required for transcription from the Epac2B promoter in the adrenal cortex, but that other mechanisms direct Epac2B expression in the endocrine pancreas.

**Figure 6:**
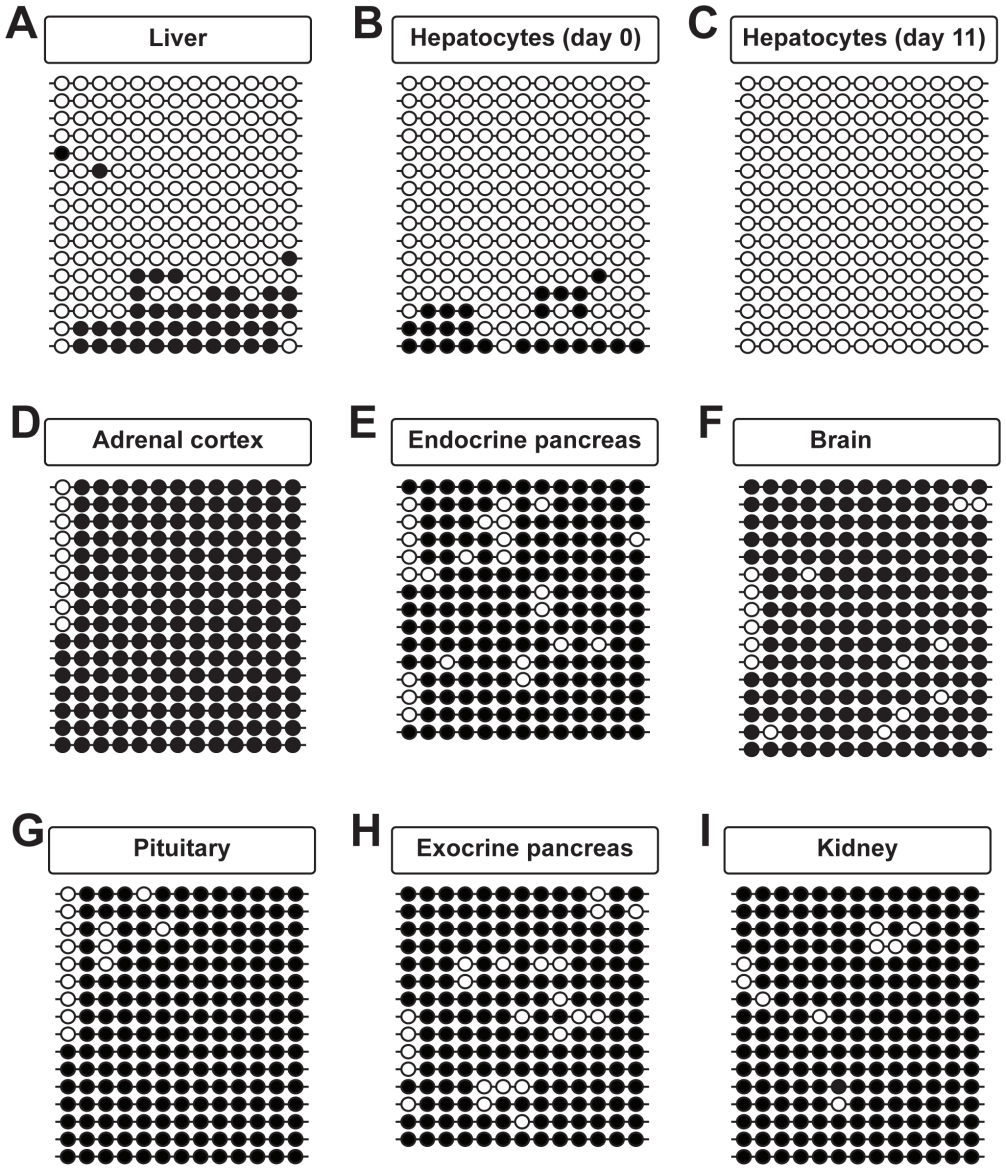
The Epac2C-promoter is hypomethylated in liver. Bisulfite sequencing analyses of the *Epac2* C-promoter (containing 13 CpG sites across a region of 469 bp, spanning nucleotides 72179672–72180140 of chr2). Genomic DNA was prepared from liver (A; 7 mice), freshly isolated hepatocytes (B; 3 mice), hepatocytes cultured for 11 days (C; 3 mice), adrenal cortex (D; 8 mice), endocrine pancreas (E; 5 mice), brain (F; 7 mice), pituitary (G; 5 mice), exocrine pancreas (H; 5 mice) and kidney (I; 7 mice). Bisulfite sequencing was performed on pooled DNA from the number of mice indicated above. The overall percentage of demethylated CpG sites and the number of clones analyzed were (number of clones in parentheses): 78% (52) in (A), 95% (48) in (B), 99.7% (64) in (C), 7% (29) in (D), 12% (15) in (E), 10% (40) in (F), 4% (28) in (G) 12% (15) in (H) and 6% (31) in (I). Each horizontal line represents one analyzed clone. Black circles: methylated CpG sites, open circles; demethylated CpG sites. Representative clones are shown for A-D, F, G and I.

To establish the methylation pattern of the putative Epac2C-promoter, primers were designed to amplify a region spanning 13 CpG sites in a region of 469 bp (figure 3A, lower panel). The Epac2C-promoter was specifically hypomethylated in the liver (figure 6A). The hypermethylated clones found in total liver probably correspond to other cell populations than the hepatocytes, like endothelial cells, Stellata and Küppfer cells [15], [16]. This interpretation is supported by analysis of DNA prepared from mouse parenchymal hepatocytes kept in culture. Bisulfite sequencing of DNA from freshly isolated hepatocytes revealed that nearly all clones were demethylated or hypomethylated (figure 6B). Upon culturing with conditions that favor hepatocyte survival, contaminating cells will disappear. In accordance with this, we observed complete demethylation of across the Epac2C promoter in hepatocytes after 11 days in culture (figure 6C). The Epac2C promoter was hypermethylated in all other tissues, both those expressing other Epac2 isoforms (*i.e.* adrenal cortex, endocrine pancreas, brain and pituitary (figure 6 D-G), and in the exocrine pancreas or kidney that does not express Epac2 (figure 6 H-I). We conclude that the 13 CpG sites of the Epac2C promoter were completely demethylated specifically in hepatocytes, which are the only cells known to express Epac2C.

## Discussion

In this study we identify promoter regions within *Epac*2 that possess the ability to drive reporter gene expression in a heterologous cell system, and establish that the regions encompassing the *Epac2B* and *Epac2C* promoters are subjected to differential DNA methylation in mouse tissues. When taken together with the transient transfection experiments (figure 3) the data strongly support the notion that transcription from *Epac2* is subjected to epigenetic control, such that a hypomethylated status allows expression of the 2B and 2C isoforms. DNA methylation patterns are generally lost when cells are kept in culture [22], and the methylation status of the *Epac2* locus was not maintained in cells cultures derived from liver, adrenal and pancreas (data not shown), with the exception of the Epac2A promoter region, which was nearly completely demethylated in all cells and tissues examined (figure 3 and data not shown). The results presented in this paper increase our understanding of how expression from the Epac2 gene is controlled, and moreover identify an additional gene that is controlled at least partly, by tissue differentially methylated regions (T-DMRs). Although many genes contain T-DMRs, relatively few have been analyzed in detail to determine the methylation status in different tissues. However, the list is constantly growing, and based on our results, *Epac2* can now be added to this group of tissue specific genes that appear to be controlled, at least partly by DNA methylation [23], [24], [25], [26], [27].

Whole genome analyses indicate that approximately 70% of annotated gene promoters are associated with a CGI, and moreover that CGI promoters generally are kept hypomethylated regardless of the transcriptional status of the gene [28]. In contrast, only around 4–8% of CGIs exhibit tissue specific methylation [19], [20], [29]. The Epac2A promoter is a typical CGI containing promoter in this respect as it was found to be hypomethylated in all cells and tissues examined independent of the expression of Epac2A. The Epac2B and Epac2C promoters fall under the category of intermediate to low CpG content promoters (ICPs, LCPs) [30]. Such promoters are often targets for tissue-dependent methylation (*i.e.* contain T-DMRs) [19], [20], [29], [31]. Moreover, and in accordance with our results on *Epac2*, T-DMRs are frequently found in promoters located in intragenic regions, and are also enriched in the proximity of alternative TSSs, suggesting that DNA methylation influences tissue specific alternative promoter usage [19], [29], [32]. Interestingly, we found the six CpG sites of the Epac2B promoter to be methylated in the endocrine pancreas despite the presence of this isoform in this tissue. In this regard, it is interesting to note that LCPs have been reported to be active even when methylated in certain cell systems [21], [33], demonstrating that a methylated status is not necessarily equal to promoter inactivation. At present we do not understand the mechanisms that integrate with DNA methylation on the Epac2B promoter, and future studies will reveal the differences in the molecular mechanisms that control expression from this promoter region in adrenocortical *vs*. pancreatic endocrine cells.

In the Epac2A promoter, CpG sites overlap with binding sites for transcription factors such as Sp1 and E2F that are enriched at CGI promoters [34]. Moreover, one CpG site co-localizes with a predicted binding site for NGFI-C in both the human murine promoters [35]. However, it remains to be experimentally tested whether these sites are of functional importance, and as is believed to be the case for other demethylated CGI-promoters, transcriptional control from this promoter is probably tightly connected to transcriptional elongation and histone modifications [28]. In fact, chromatin immunoprecipitation (ChIP) data available via the Encyclopedia of DNA Elements (ENCODE) consortium demonstrate that histone marks associated with active promoters (H3K27ac and H3K4me3) [36], [37], as well as RNA polymerase II, are enriched across the Epac2A promoter in brain compared to liver and kidney, whereas H3K27me3, which is associated with gene repression [38] is enriched in liver and kidney compared to brain [39].

In the Epac2C promoter, three CpG sites are located at corresponding positions in the murine and human promoters, but none of these overlap with predicted transcription factor binding sites. However, of potential interest is that a CpG site in the murine promoter is part of a recognition sequence for the nuclear receptor LXR that is known to have several functions in this organ [40]. There is only one CpG site in the Epac2B promoter that is positioned at corresponding sites in the murine and human promoters. This site co-localizes with potential binding elements for ubiquitous transcription factors (i.e. for CBF, CETS1p54, ELK1, CP2, NRF2 and STAT3). Taken together, these findings probably indicate that DNA methylation represses Epac2B and 2C promoter activity by affecting chromatin architecture rather than inhibiting the binding of specific transcription factors. This notion is also supported by that both the GC content and the number of CpG-sites is conserved between mouse and human in the Epac2B and 2C promoters, but that the precise location of each CpG site is not (data not shown).

As described in the introduction, the Epac2 isoforms exhibit a strict tissue specific expression pattern. The results presented in figure 2 are generally in agreement with published literature on Epac2 expression in mice [5], [6], [8]. Importantly, however, the identification of Epac2B in endocrine pancreas is novel. The endocrine pancreas thus differs from the other tissues examined as it expresses similar amounts of two Epac2 isoforms (Epac2A and Epac2B) (figure 2C). Since the alpha and beta cells compose about 85–90% of the endocrine pancreas cell mass, both Epac2B and Epac2A are likely to be present in one or both of these cell types. The potential functional significance of two Epac2 isoforms in the same cells or tissue is to be determined, but this finding may have implications for experiments designed to determine the roles of Epac2 in insulin secretion.

At present little is known about the functional differences of the Epac2 isoforms and about the physiological significance for their restricted expression patterns. The three domains that differ between Epac2A, Epac2B and Epac2C (cAMP-A, DEP and RA domains, see figure 1A) have all been implicated in sub-cellular localization of Epac2 [1], suggesting that the isoforms might be directed to different compartments that specify their functions. In line with this, overexpression of Epac2A in MIN6 cells (the endogenous isoform in this ß-cell line), amplifies the secretory response to combined stimulation with glucose and cAMP. Interestingly, Epac2B can not replace Epac2A in this experimental set-up [5]. This functional difference is proposed to reflect a critical role of the cAMP-A domain present in Epac2A for directing the protein to the plasma membrane, whereas Epac2B, which lacks this domain, is diffusely spread in the cytoplasm [5]. Epac2B is expressed in steroidogenic cells [5], [7]. In adrenocortical cells, Epac2B is localized to the nucleus or the nuclear envelope [7], and it is interesting to note that Epac1, which contains the same protein domains as Epac2B, is also localized in connection with the nucleus [41], [42]. The potential roles of Epac2B in the nucleus are still unknown, but Epac1 has been associated with nuclear functions, such as trafficking of the DNA-damage responsive protein kinase [41] and interaction with the nuclear pore complex through RanBP2 [43]. Epac2C lacks the DEP-domain, but contains the RA domains shared by all Epac proteins (figure 1A). This domain anchors Epac2 to Ras-containing membranes, and thereby couples cAMP- and Ras-signaling in Rap activation [44], [45]. The RA-domain of Epac1 does not have this ability, and Epac1 depends on its DEP domain for membrane targeting [46].

In summary, this study demonstrates the importance of epigenetic mechanisms in the spatial control of Epac2 expression. It confirms that the different Epac2 isoforms arise by alternative promoter usage, and strongly indicate that TDMRs located in the intragenic promoters contribute in the control of isoform expression. Tissue specific transcriptional regulators most likely work together with epigenetic marks to achieve the strictly controlled transcription from *Epac2*.

## Materials and Methods

### Mice

All mouse tissues used in this study were derived from the C57BL/6J strain (male and female). Mice were kept under a 12h light/dark cycle with food and water *ad libitum*. Animals were killed by CO_2_ prior to cell- and tissue harvesting. For hepatocyte isolation, mice were anesthetized with isoflurane/oxygen. The projects and procedures for animal handling were approved by ethical committees and conducted in accordance with the Norwegian and Swedish legislation and regulations governing experiments using live animals in Norway and Sweden, respectively. The Animal Care and Use Programs at University of Bergen is accredited by AAALAC international. Permit numbers: 20113453,University of Bergen; Uppsala ethical committee C253/10, Uppsala University.

### Isolation of Mouse Hepatocytes

Primary hepatocytes were isolated from C57BL/6 mice. A venous cannula perforated the hepatic portal vein to give access to the mouse’s blood supply. A tube was connected to the cannula, and EBSS (100 μl; 500 μM EGTA, 100 U/ml penicillin, 100 μg/ml streptomycin) was infused into the vein at 3,8 ml/min. To avoid accumulation of fluid the inferior vena cava was cut immediately after the infusion started. After 20 min the perfusion solution was changed to EBSS (100 ml) containing CaCl_2_ (2M) and collagenase (300 μg/ml) for 25 minutes at 3,8 ml/min. The liver was excised and incubated in Williams E Medium I (VME I) containing 100 U/ml penicillin, 100 μg/ml streptomycin and FBS (10%) to release the hepatocytes. All subsequent steps were performed on ice or at 4^°^C. The solution was filtered through a 40 μm grid, pelleted at 50×g for 5 min, and resuspended in WME I (20 ml). The cell suspension was transferred to a tube containing 1∶1 WME I and Percoll (20 ml) and centrifuged at 750×g for 5 min. The cells were washed twice in WME I (40 ml) at 50×g, 5 minutes. Finally the cells were resuspended in WME I (20 ml). Cells were seeded on 6- well plates pre-coated with collagen. 3×10^5^ cells were seeded into WME I (2 ml)/well, after one wash with PBS. After 4 hours, the medium was changed to WME II (containing 100 U/ml penicillin, 100 μg/ml streptomycin, ITS+ and dexamethasone (0.1 μM).

### Isolation of Genomic DNA

Genomic DNA from mouse tissues, paraffin sections or primary cells was isolated employing the QIAGEN DNeasy kit (Gaithersburg, USA) following the manufacturer’s instructions.

### RNA Isolation and Reverse Transcriptase PCR (RT-PCR)

RNA was isolated from materials pre-stored at RNA-later buffer, using the RNeasy Protect Mini Kit (QIAGEN, Gaithersburg, USA). One μg of total RNA was employed for the cDNA synthesis, performed with the iScript cDNA synthesis kit from BioRad (California, USA). RT-PCR was performed with 20 ng cDNA template and GoFlexi DNA polymerase (Promega, Madison WI, USA) on a MJ Research PTC-200 under the following conditions: initialization at 96°C for 2,5 min and 33 cycles of 30s at 96°C, 25s at 59°C and 30s at 72°C, and a final elongation step for 10 min at 72°C (see table 1 for primers). RT-PCR products were submitted to 2.3% agarose gel electrophoresis, and subsequently sequenced to verify transcript isoform subtype.

### Preparation Protein Extracts

Tissue extracts were prepared by lysis in Laemmli buffer (5% SDS, 62 mM Tris-HCl (pH6.8), 3 mM EDTA and 10% glycerol, supplemented with complete protease inhibitor cocktail EDTA-free, Roche Applied Science). As positive controls for the Epac2 isoforms in the immunoblotting experiments, Cos-1 cells were transfected with flag-tagged Epac2A/Epac2B/Epac2C expression vectors (pFLAG/CMV2; Sigma) using Fugene 6 (Roche Applied Science). Whole-cell extracts were prepared by lysis in RIPA buffer (50 mM Tris-HCl (pH 8.0), 150 mM NaCl, 0.5% deoxycholate, 1% Nonidet P-40, 0.1% SDS, 1 mM phe-nylmethylsulfonyl fluoride, 2 mM sodium orthovanadate, and 2 µg/ml aprotinin and leupeptin).

### Isolation of Exocrine and Endocrine Cells from the Mouse Pancreas

C57BL/6J mice were killed by decapitation in deep CO_2_ anesthesia_;_ the pancreas was dissected out and kept on ice in buffer (125 mM NaCl, 4.8 mM KCl, 1.28 mM CaCl_2_, 1.2 mM MgCl_2_ and 25 mM HEPES, adjusted to pH 7.4 with NaOH). The tissue was then injected with collagenase P and cut into 3–4 pieces before digestion in a shaking water bath (37°C) for 7 minutes. The collagenase digestion was terminated by addition of BSA (0.1 g/mL), and the remaining tissue was thoroughly washed with ice-cold buffer. Islets of Langerhans were then separated from exocrine tissue by handpicking with a pipette under a stereomicroscope. Isolated islets and exocrine tissue, were immediately put on RNA-later (Sigma) for storage before RNA or DNA isolation, or frozen at −80°C for protein extraction.

### Immunoblotting

Protein extracts (250–300 μg of tissue extracts, 20 μg of Cos1-cell extract with overexpressed Epac2 isoforms) were separated by SDS-PAGE and transferred to nitrocellulose membranes. The membranes were blocked with I-block and incubated overnight with primary antibodies and subsequently with anti-mouse antibodies for 1h at RT. Antibody dilutions were 1∶100 for the anti-Epac2 antibody (5B1, Cell Signaling), whereas anti-actin (ab-6276; Abcam, Cambridge, UK) and anti-mouse horseradish peroxidase (HRP)-conjugated antibodies (Santa Cruz Biotechnology) were used at 1∶10000. Chemiluminescence signals were developed by the SuperSignal West Pico chemiluminescent substrate (Pierce Biotechnology).

### Construction and *in vitro* Methylation of Reporter Plasmids

Fragments corresponding to the putative Epac2 promoter regions were amplified by PCR with primers as shown in table 2 (Phusion High-Fidelity DNA polymerase, New England Biolabs, United Kingdom), and subcloned upstream of the luciferase reporter gene in a CpG-free vector (pCpG-basic; provided by Dr. M. Klug, Regensburg University Hospital, Germany) to create the reporter plasmids pCpG-Epac2A, pCpG-Epac2B and pCpG-Epac2C. Detailed information about the plasmid constructions will be provided upon request. The analogous cytomegalus (CMV)-enhancer/elongation factor 1 (EF) promoter containing vector pCpG/CMV-EF1alpha was employed as a positive promoter control (also named pCpGfree-mcs, InVivogen, San Diego, USA). The reporter plasmids were methylated *in vitro* by using the *Sss*I CpG methylase (New England Biolabs, United Kingdom). Plasmid DNA was incubated at 37°C for four hours with *Sss*I (2 U/μg DNA) in presence of S-adenosylmethionine (SAM) (160 μM), with an addition of extra *Sss*I (0.3 U/μg DNA) and SAM (160 μM) after two hours. The unmethylated control plasmids were treated as above, but in the absence of *Sss*I.

**Table 2:** Overview of primers employed in sodium bisulfite sequencing assays (PCR).

Target region	Nested primer localization	Direction	Sequence (5′–3′)	PCR Size (bp)
Epac2A	“Outer”	F	AGGAGGATTAATGGGTTTTTAAGGAAA	502
		R	AAATAACACCCCAC**R**AATAAACCC	
	“Inner”	F	GAGAGGTAGTAGTTATTTTAGTGGTATTTGTTGTA	441
		R	CAAATCCC**R**CACCCCACC	
Epac2B	“Outer”	F	TGTTTTTTTTTTTATAGTTTTGATTTTTTTAGATT	492
		R	TCCCCAATCTCCCACCCTTC	
	“Inner”	F	GGAAGTTTGTTTTAATGATTTGGATTTG	438
		R	CCTTCACCAAAAAATCAAAAACCC	
Epac2C	“Outer”	F	TAGTGTGTAAGTTTTATAAAAGTTGGTAGGTGTT	522
		R	TATCCCTCCCCAAAATCCCTACA	
	“Inner”	F	TGGTAGGTGTTTAGTAGGAGGTTTAGAAAG	469
		R	AAACCAACCAAATAAAATACTCCTAACTCTC	

**R** is a A or G nucleotide.

### Transfection of Cells and Luciferase Reporter Assay

Transformed African Green Monkey Kidney Fibroblast Cells (Cos-1) were plated in 24-well plates and transiently transfected the following day using FuGENE HD (Roche Diagnostic, Germany). The cells were transfected with reporter plasmids (200 ng/well), and 48 h later, the cells were lysed in luciferase buffer (10 mM Tris-HCl pH 8, 150 mM NaCl, 0.65% NP-40, 4 mM EDTA). Luciferase activities were measured in total cell extract using the Luciferase Assay Kit A (BIO Thema AB, Dalarö, Sweden) according to manufacturer’s protocol on a LUCY-3 luminometer (Anthos, Austria).

### Laser Dissection of Paraffin Embedded Mouse Tissues

Laser dissections were performed with a system comprised by an inverted microscope (Axiovert 200; Zeiss, New York, NY) and an air-cooled nitrogen laser (model VSL-37 ND-S) from P.A.L.M. Microlaser Technologies GmbH (Munich, Germany). Micro-dissection was performed on 7-μm-thick paraffin sections of mouse adrenal glands. Paraffin embedding and sectioning were performed according to standard protocols.

### Sodium Bisulfite Sequencing

Genomic DNA (5–500 ng) was subjected to sodium bisulfite modification with the EZ DNA Methylation-Gold kit (Zymo Research, Orange, CA) according to the producer’s instruction. PCR amplification was carried out using primers (see table 3) specific for the bisulfite-converted target DNA in a nested PCR-strategy, employing GoFlexi DNA polymerase (Promega, Madison WI, USA) on a MJ Research PTC-200 (BioRad, California, USA). The PCR amplicons were subcloned into the pGEM-T-easy vector (Promega, Madison, WI) after purification (QIAEX II Gel Extraction Kit, Gaithersburg, USA) and subsequently sequenced and with T7 or Sp6 primers employing BigDye terminator 3.1 kit on the Applied Biosystems 3730XL Analyzer. Sequence data were then investigated by Sequence Scanner software (Applied Biosystems), alignments performed with ClustalX and CpG-site diagrams were made using Adobe Illustrator software (Adobe).

**Table 3:** PCR primers for construction of reporter plasmids (pCpG-constructs).

Target promoter region	Direction	Sequence (5′ –3′)	RE	PCR Size (bp)	Chromosome start/end position (mm10/chr 2)
Epac2A	F	CTAT***ACTAGT***AAGCCTTCCCAGCCCTGCC	*Spe*I	763	71980816
	R	TAGT***CCATGGCCCGGG***CTCGCATGTATGTACCCTCCCTG	*Nco*I,*Xma*I		71981578
Epac2B	F	CTAT***ACTAGT***AATCTCGCAAACCACTGTGACTAAG	*Spe*I	946	72054067
	R	CATTA***GGATCC***GCGTCTGTTGCGGGAAGTTCT	*Bam*HI		72055012
Epac2C	F	CTAT***ACTAGT***AAGAAAATGACCCCAATTTCCTTCA	*Spe*I	648	72179380
	R	TAGT***CCATGG***CATCACACTCCTTCTTCTCTTCCTCAG	*Nco*I		72180027

## Supporting Information

Figure S1 **Alignment of mouse Epac2 protein isoforms.** Full amino acid sequence (aa) is shown for each of the Epac2 isoforms; Epac2A1 (canonical sequence), Epac2A2, Epac2B and Epac2C. Functional domains are shown colored; cAMP-A and B; cAMP-binding domain A (red) and B (green), DEP; Dishevelled/Egl–10/Plekstrin domain (yellow), REM; Ras exchange motif domain (blue), RA; Ras association domain (orange), RasGEF; Guanine nucleotide exchange factor (GEF) for Ras-like small GTPases (pink). Stars below sequence alignment indicate full aa identity (100%) among all four Epac2 isoforms. Note the dissimilarity at AA 145/147 (of canonical sequence) between Epac2A and Epac2B due to translation from Exon1b for the Epac2B isoform (grey box). Also, note the lack of the 18 aa peptide in Epac2A2, compared to Epac2A1/Epac2B (corresponding to aa 180–217 of canonical sequence). Alignment was produced by ClustalX (1.83), and modified in GeneDoc by employing the following mouse protein sequences; Epac2A1; A2ASW4, Epac2A2; A2ASW3, Epac2B; A2ASW8 and Epac2C; Q9EQZ6-2.
(TIF)

Figure S2 **rVISTA evolutionary conserved genome alignment.** rVISTA genome alignment of evolutionary conserved regions (ECR) between the human hg19-genome and the mouse mm10 base genome was performed on the three Epac2 promoter regions: A; Epac2A promoter (chr2 71980880–71981553, B; Epac2B promoter (chr2 72054237–72054766), C; Epac2C promoter (chr2 72179738–72180207).
(TIF)

Figure S3 ***In silico*** **prediction of CpG islands in** ***Epac2*** **.** The sequence corresponding to *Epac2*, expanded by 3 kb upstream and downstream of the gene, was submitted to EMBOSS for CpG-Island (CGI) prediction (http://www.ebi.ac.uk/Tools/emboss/cpgplot/). Only one CGI, containing 60 CpG-sites over 548bp, was predicted in this sequence. The CGI overlaps with the Epac2A promoter region. For clarity, a region of 1000bp covering the CGI is shown. Default settings were employed in analysis. A; Calculated observed/expected ratio of CpG patterns (Obs/Exp-value). B; Calculated percent G’s+percent C’s in the region (Percent C+G content); C; The resulting CGI-region identified, based on A and B. For further details see website documentation and original publication by Gardiner-Garden & Frommer [18].
(TIF)
