# Supplementary figures and images for: DNA Methylation of Alternative Promoters Directs Tissue Specific Expression of Epac2 Isoforms
Source: PLoS One. 2013 Jul 4;8(7):e67925. doi: 10.1371/journal.pone.0067925 (PMC3701594; doi:10.1371/journal.pone.0067925)

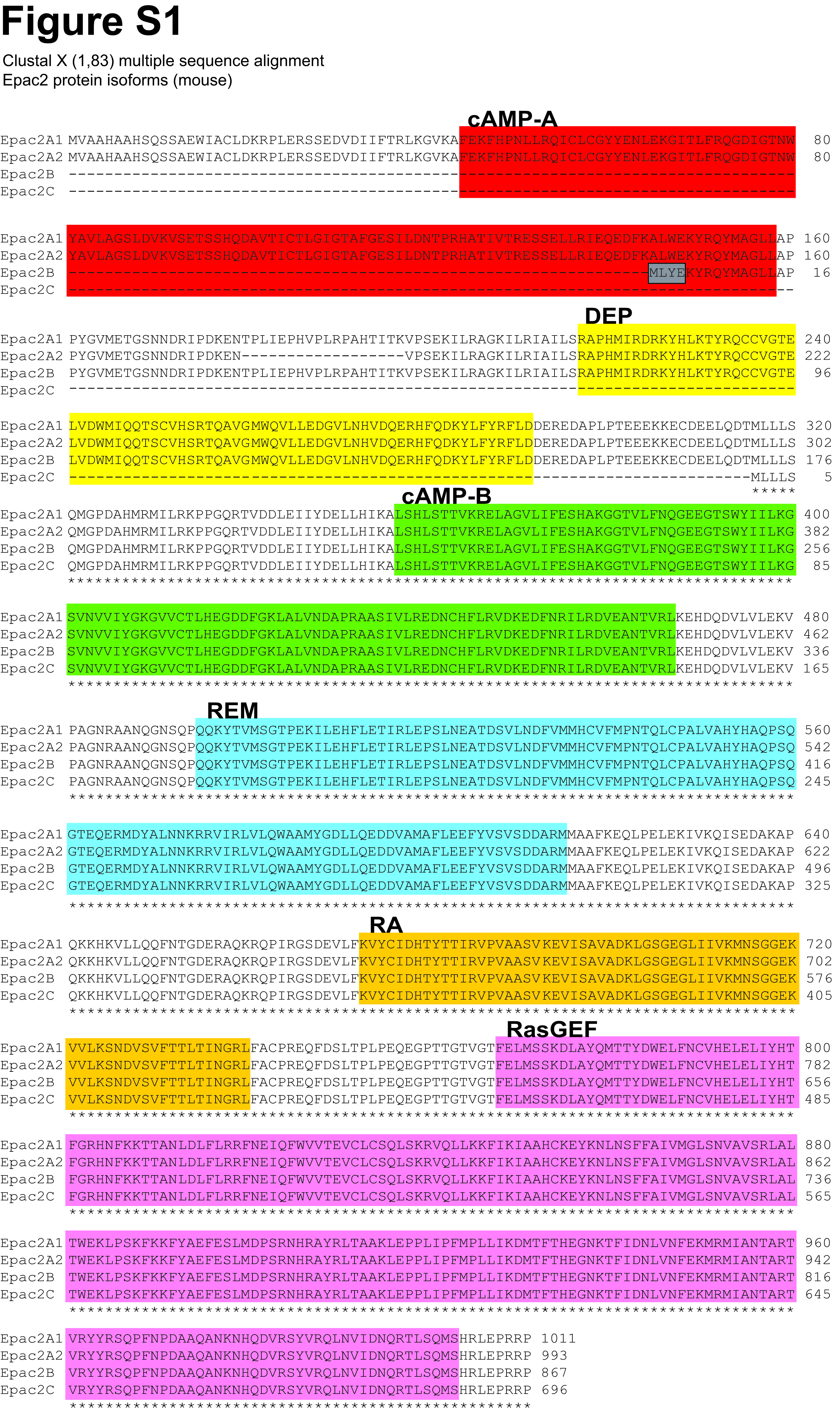

Supplement: Figure S1 — Alignment of mouse Epac2 protein isoforms. Full amino acid sequence (aa) is shown for each of the Epac2 isoforms; Epac2A1 (canonical sequence), Epac2A2, Epac2B and Epac2C. Functional domains are shown colored; cAMP-A and B; cAMP-binding domain A (red) and B (green), DEP; Dishevelled/Egl–10/Plekstrin domain (yellow), REM; Ras exchange motif domain (blue), RA; Ras association domain (orange), RasGEF; Guanine nucleotide exchange factor (GEF) for Ras-like small GTPases (pink). Stars below sequence alignment indicate full aa identity (100%) among all four Epac2 isoforms. Note the dissimilarity at AA 145/147 (of canonical sequence) between Epac2A and Epac2B due to translation from Exon1b for the Epac2B isoform (grey box). Also, note the lack of the 18 aa peptide in Epac2A2, compared to Epac2A1/Epac2B (corresponding to aa 180–217 of canonical sequence). Alignment was produced by ClustalX (1.83), and modified in GeneDoc by employing the following mouse protein sequences; Epac2A1; A2ASW4, Epac2A2; A2ASW3, Epac2B; A2ASW8 and Epac2C; Q9EQZ6-2. (TIF) [file pone.0067925.s001.tif]

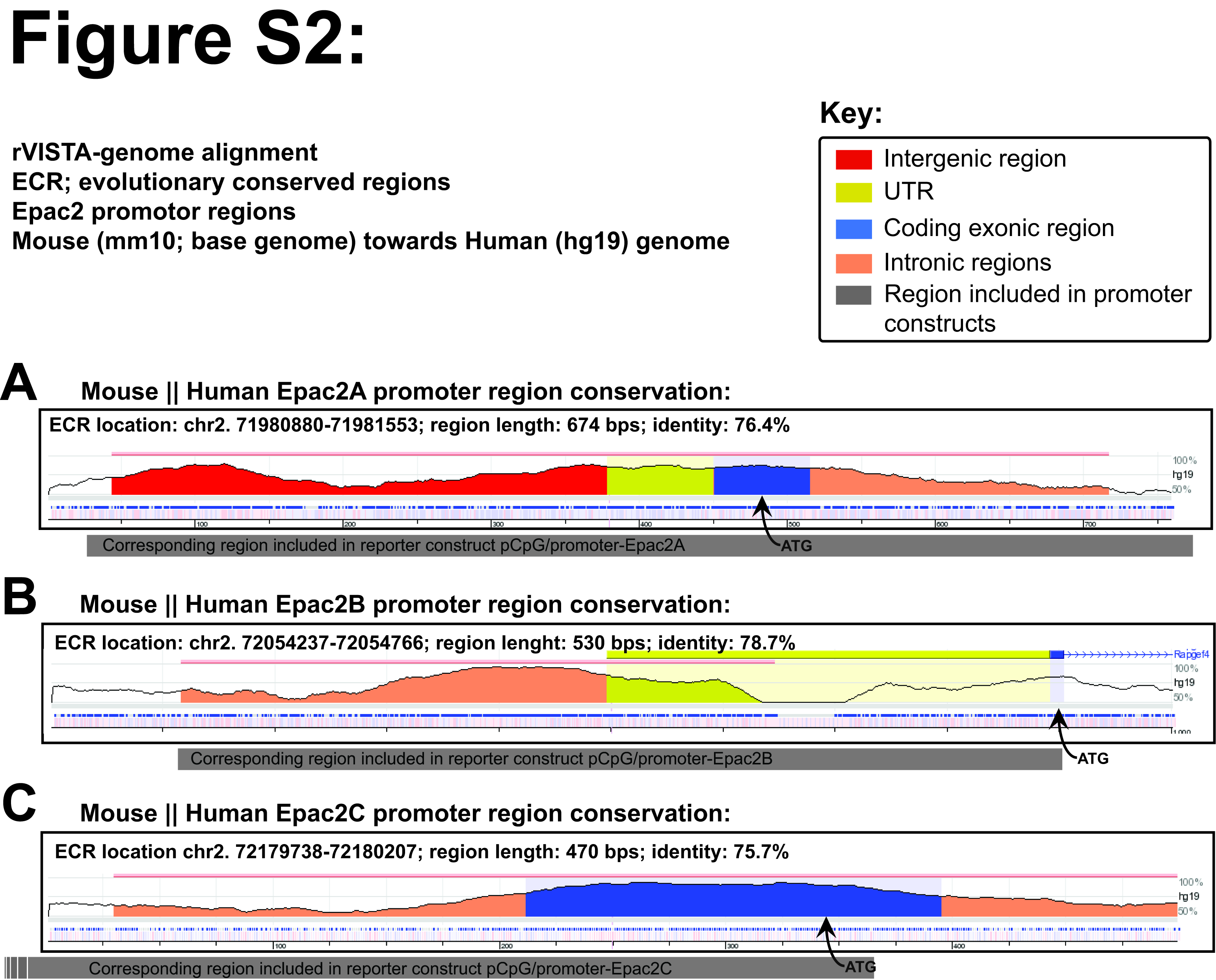

Supplement: Figure S2 — rVISTA evolutionary conserved genome alignment. rVISTA genome alignment of evolutionary conserved regions (ECR) between the human hg19-genome and the mouse mm10 base genome was performed on the three Epac2 promoter regions: A; Epac2A promoter (chr2 71980880–71981553, B; Epac2B promoter (chr2 72054237–72054766), C; Epac2C promoter (chr2 72179738–72180207). (TIF) [file pone.0067925.s002.tif]

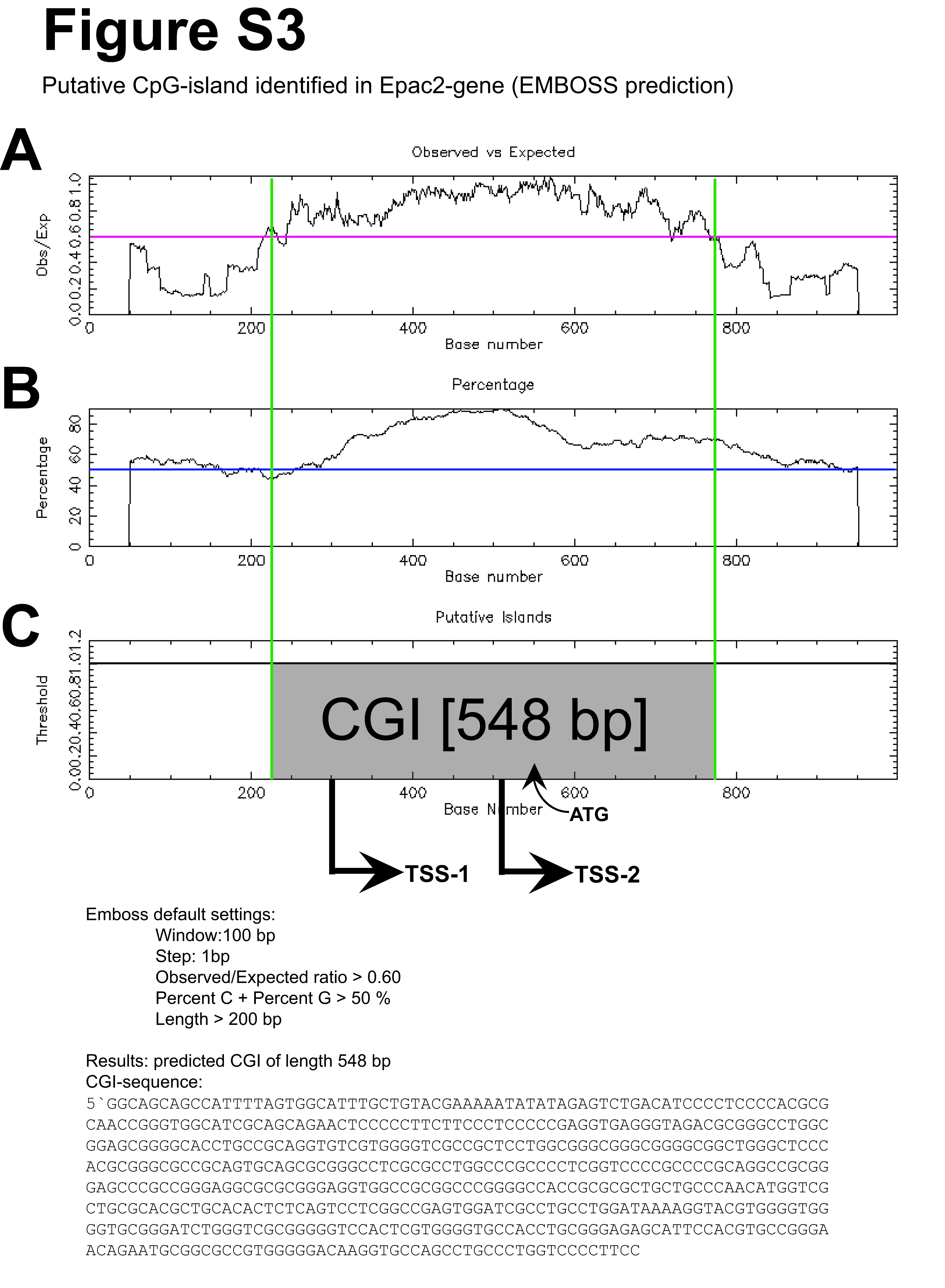

Supplement: Figure S3 — In silico prediction of CpG islands in Epac2 . The sequence corresponding to Epac2, expanded by 3 kb upstream and downstream of the gene, was submitted to EMBOSS for CpG-Island (CGI) prediction (http://www.ebi.ac.uk/Tools/emboss/cpgplot/). Only one CGI, containing 60 CpG-sites over 548bp, was predicted in this sequence. The CGI overlaps with the Epac2A promoter region. For clarity, a region of 1000bp covering the CGI is shown. Default settings were employed in analysis. A; Calculated observed/expected ratio of CpG patterns (Obs/Exp-value). B; Calculated percent G’s+percent C’s in the region (Percent C+G content); C; The resulting CGI-region identified, based on A and B. For further details see website documentation and original publication by Gardiner-Garden & Frommer [18]. (TIF) [file pone.0067925.s003.tif]
